# Code-reading support environment visualizing three fields and educational practice to understand nested loops

**DOI:** 10.1186/s41039-016-0027-3

**Published:** 2016-01-13

**Authors:** Koichi Yamashita, Takamasa Nagao, Satoru Kogure, Yasuhiro Noguchi, Tatsuhiro Konishi, Yukihiro Itoh

**Affiliations:** 1 Faculty of Business Administration, Tokoha University, 1230 Miyakoda, Kita-ku, Hamamatsu, Shizuoka 431-2102 Japan; 2 Graduate School of Informatics, Shizuoka University, 3-5-1 Johoku, Naka-ku, Hamamatsu, Shizuoka 432-8011 Japan; 3 Faculty of Informatics, Shizuoka University, 3-5-1 Johoku, Naka-ku, Hamamatsu, Shizuoka 432-8011 Japan; 4 Shizuoka University, 3-5-1 Johoku, Naka-ku, Hamamatsu, Shizuoka 432-8011 Japan

**Keywords:** Education for programming, Domain world models, Learning environment for exercise, Classroom practice, Learning by code reading

## Abstract

In this paper, we describe a code-reading support environment and practical classroom applications using this environment to understand nested loops. Previously, we developed a code-reading support system based on visualization of the relationships among the program code, target domain world, and operations. We implemented the proposed system in exercises with nested loops. The evaluation results suggested that students could frequently fulfill learning objectives using the proposed system. However, we also discovered that some students experienced a learning impasse in the classroom. We attempted to address these students with two supporting approaches: bridging the gap between the generalization structures in the program code and their corresponding operations and enabling learners to predict the behavior of the nested loops. In this paper, we extend our previous system with new functions based on our two supporting approaches. Further, we implement the system in another classroom for nested loops. We describe a correlation between the proposed system and an understanding of nested loops using pre-/post-test comparisons. We discuss how code reading using the proposed system allows learners to cultivate a superior understanding of the program code.

## Introduction

With the continued rapid development and spread of information equipment such as smartphones, productivity improvements for program codes have been increasingly required. Software-developing environments have been enhanced, and programming languages have developed their descriptive capabilities. Programming skills are becoming necessary for not only software engineers. In the context of society informatization, increasing numbers of learners require an education in computer programming (Robins et al. 2003; Pears et al. 2007; Konecki & Petrlić 2014).

For several years, we have conducted programming classes for a wide variety of students. Through our experience in the classroom, we have paid attention to three fundamental skills that novice programming students tend to find difficult to acquire:

- Control structure can be and often is nested inside of another one. Students frequently have difficulty understanding nested structures in control flows and code descriptions. (Nesting)
- Students often struggle to generalize a set of concrete operations into an abstract function with variables. They tend to be able to interpret the processing contents of the first lap of the loop, the second lap of that, the third lap of that, and so on, while not to the contents of the *n*th lap of the loop. (Generalization)
- Students sometimes fail to grasp how the values of variables change with the execution of each statement. (Tracing; we treat tracing as tracing values of variables used in the code.)

Nested loops are a learning target with which novice learners frequently have an initial difficulty. This is because to fully comprehend this concept requires that the learner understands all three of the abovementioned fundamentals. Koppelman and van Dijk (2010) emphasized the importance of nested loops as one of the targets required to understand the concept of abstraction. However, limited exposure in programming courses constrains the efforts of learners to develop a thorough understanding of these fundamental concepts. The purpose of our study is to encourage students to learn these concepts efficiently. We have introduced learning support systems into classroom exercises in nested loops for several years (Kogure et al. 2013).

In this paper, we describe our code-reading support environment and the classroom applications of using this environment to understand nested loops. Previously, we developed a learning support system for code reading (Kogure et al. 2012). As in our previous work, we assume that learners will understand programs and algorithms by recalling an image consisting of three fields: the program code, objects processed by the program (i.e., the target domain world), and a sequence of concrete operations for the target domain. Learners must comprehend the relationships and correspondence among the components in each field. Many existing systems intuitively visualize the target domain world and reproduce the transition of its status. However, they do not have functions to visualize the correspondence between the program code and the concrete operations. The proposed system visualizes the three fields and their relationships to support understanding the relationships and correspondence among their components.

In our previous work (Kogure et al. 2013), we conducted exercise classes implementing our system to assist the understanding of nested loops and determined that some students experienced a learning impasse in the classroom. Based on the implicit and explicit feedback in our previous classroom practices, we constructed two approaches to cope with the impasse: bridging the gap between generalization structures in the program code and their corresponding operations and enabling learners to predict the behavior of a nested loop by observing two characteristics in the code.

In this paper, we present an overview of our previous system, new support strategies and functions that have been incorporated in the proposed system, and renewed classroom practice in helping students understand nested loops. We describe our previous learning support system, the two approaches to avoid the learning impasse, and the proposed extended system in the “Learning environment for programs and algorithms” section. We provide an overview of our classroom practice, our controlled experiment, and the evaluation results of the proposed system in the “Educational practice” section. Our evaluation results suggest that there is a correlation between our extended system and an understanding of nested loops. In the “Related works” section, we clarify the position of the proposed system, citing representative works. We discuss how code reading with the proposed system supports learners to cultivate an improved understanding of the program code and how the proposed system contributes to the overall learning of programming in the “Discussion” section. We conclude with a brief summary and discussion of future work in the “Conclusion” section.

## Learning environment for programs and algorithms

### Our previous work

**Fig. 1:**
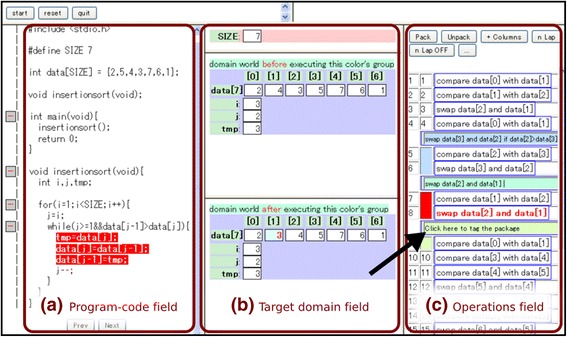
Overview of the LEPA environment, which has the program code field visualized in (**a**), target domain field in (**b**), and operation field in (**c**).

In our previous work (Kogure et al. 2012), we assumed that learners require an image consisting of three fields and understand the relationships among their components. The three fields are the program code field, target domain field, and operation field. With this assumption, we developed a system called learning environment for programs and algorithms (LEPA) that supported the learner in understanding programs. Figure 1 presents an overview of the learning support environment provided by LEPA. The three fields are reproduced in (a), (b), and (c).

LEPA is a code-reading support system for a typical program code in the C language in programming education for novice learners. Given a C code, unless the input code includes syntax errors and/or runtime errors, LEPA automatically generates a browser-based environment supporting learners to read the code. Consequently, it is not intended that learners use LEPA as a code-developing environment. LEPA provides learners with an environment to read an example code fragment provided by a teacher or textbook.

When a learner clicks on any of the operations in (c), the system displays the state of the target domain after executing the operation in (b). Thus, the learner can understand the role of a certain sequence of operations by comparing or observing the visualized target domain field before and after execution. Furthermore, the system highlights the code fragment in (a) corresponding to the selected operation in (c) and vice versa when the learner first clicks on a fragment in (a). The correspondence between a code fragment and an operation is therefore apparent to the learner. We believe that important information can be understood and retained using LEPA’s visualization, including what happens in the target domain field when a concrete operation is executed and what kind of code is required to create this effect.

Learning with LEPA is based on the learner’s externalization of what they have observed. Externalization is demonstrated with a process of packing and tagging using the GUI interface. The term “packing” in this paper means grouping a certain sequence of operations with single abstract function into a package. If such sequence of operations is discovered in (c), then the following two options are available:

1. The learner can push the “pack” button to pack the selected operation sequence into a package. Packages are permitted in a nested structure.
2. The learner can tag the package with a natural-language description according to its function (as indicated by the arrow in Fig. 1).

The resulting packed structure of the operation sequence ideally comes closer to the program code’s structure. The learner will arrive at an understanding of the entire control sequence for the program code by completing a series of these activities.

**Fig. 2:**
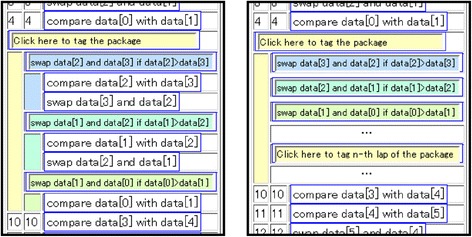
Tagging a package and tagging the *n*th lap of a package

Externalization activities can be classified into two groups according to the repetitiveness in the target operations: abstraction for operations that are not repetitive and generalization for operations that are repetitive. LEPA supports the former activity with packing operations and the latter by tagging the *n*th lap of a loop (as indicated in Fig. 2).

### Extending approach

In our previous work (Kogure et al. 2013), we implemented LEPA in exercise with nested loops. In these classes, we discovered that some students experienced a learning impasse. Based on the score differences between pre- and post-tests, the system did not indicate effects on the learner’s ability to generalize. To address this impasse, we now extend LEPA based on two supporting approaches. First, we consider that a factor of the impasse was the gap between the generalization structures in the program code and in the corresponding operations. Therefore, we extend LEPA to support learners to bridge the gap. Then, we consider that a reduced ability to grasp the relationship between a code fragment and behavior hindered an improved understanding of nested loops. Therefore, we also extend LEPA to support learners to predict the behavior of a nested loop based on its classification derived by two characteristics in the code. In this section, we describe these two approaches. Hereafter, we call our previous system described in (Kogure et al. 2012) LEPA1 and the proposed extended system LEPA2.

#### Gap between the representations of generalization

**Fig. 3:**
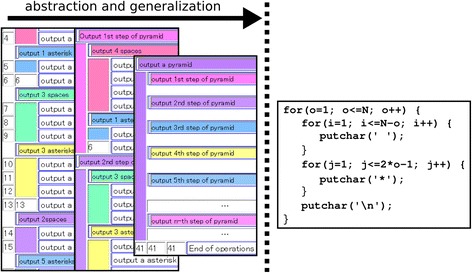
Gap between the generalized operations and the program code

Packing operations brings their structure closer to the program code. However, a gap can be found between the structure of a package and the corresponding fragment of the program code, as illustrated in Fig. 3.

The right side in Fig. 3 presents the program code that displays an *N*-step pyramid by outputting an appropriate number of spaces, asterisks, and new lines. The left side indicates the transition of the package structure corresponding to the code. It is our opinion that this is the most plausible transition.

We assume that these packages are created with the following steps in terms of the learner’s learning trajectory. First, the learner observes three fields and consequently creates two packages such as “output *x* spaces” and “output *y* asterisks.” In this case, the learner assigns concrete numerals for *x* and *y*, rather than variables. Then, they are packed hierarchically with an operation that outputs a new line that is tagged “output *z*th step of the pyramid.” At this stage, *z* is also a concrete numeral. The learner continues these steps until all of the operations in the operation field are included in the packages. Finally, *N* packages are acquired, packed, and tagged for the *n*th lap.

In LEPA1, the target for generalized tagging is only available at the first hierarchical level. This means that the learner tags the *n*th lap with variables that are exclusively from the package, “output *z*th step of the pyramid.” LEPA1 hides any levels that are deeper than this in the operation field (i.e., outputting spaces, asterisks, and new lines). However, the program code for a nested loop is provided throughout the hierarchy. Having generalized the first-level hierarchy into “output *k*th step of the pyramid” with the variable *k*, the learner moves on to the deeper levels such as “output *N*-*k* spaces” or “output 2*k*-1 asterisks.” Given generalizations throughout the hierarchy, the learner can realize the meaning of the control statements for the nested loops in the program code.

Based on these discussions, we have implemented a function that enables users to generalize a package while retaining the explicit hierarchy. Thus, the learner can tag the *n*th lap of the inner loops. Further, we have implemented a function that automatically generates a template for the general tag with the following steps:

1. Discriminating variable words from invariable words in the set of tags on the packages to be generalized
2. Replacing variable words with a series of symbols such as “_”

**Fig. 4:**
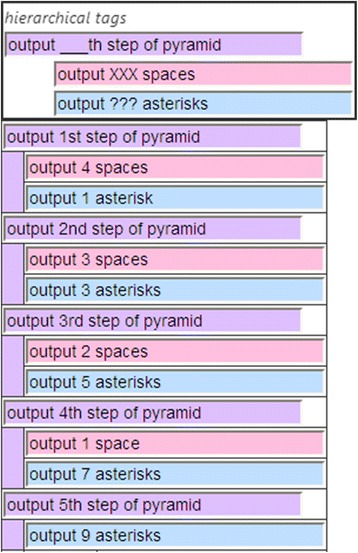
Generalized packages while keeping the hierarchy explicit

For example, in displaying a pyramid, the proposed system generates templates such as “output XXX spaces,” “output ??? asterisks,” and “output ___th step of the pyramid” from the set of tags. By providing these templates, the proposed system encourages learners to formulate “XXX,” “???,” and “___” with loop control variables in the program code. The screenshot in Fig. 4 depicts the implemented functions. They are intended to assist learners to understand the structure of nested loops.

#### Two characteristics in nested loop code

Programmers implement loops to include both identical operations and a wide variety of repetitive operations by appropriately referencing the loop’s control variables. For example, different values are displayed by iterative execution of a statement to display the value of the variable *i* in a loop controlled by *i*. Therefore, the iteration package corresponding to the statement consists of a sequence of operations with different descriptions. Generalization in the proposed system involves two phases: identifying operations that regularly vary in the iterative package of the operation field and formulating this regularity. Nested loops increase the diversity of repetitiveness in the corresponding operations. We must consider not only the inner and outer loops where the control variables are referenced but also loops with references to variables that control the outer loop. This diversity can be regarded as a factor that novice programmers tend to find difficult to comprehend the relationship between the code of a nested loop and its behavior.

We constructed the second supporting approach to understand relationships by developing a classification of nested loops. By investigating textbooks and exercises, we have focused on the following two characteristics in nested loop code:

- Ch1. A statement to iterate in the inner loop references control variables from the outer loop.
- Ch2. A conditional statement in the inner loop references control variables from the outer loop.

**Fig. 5:**
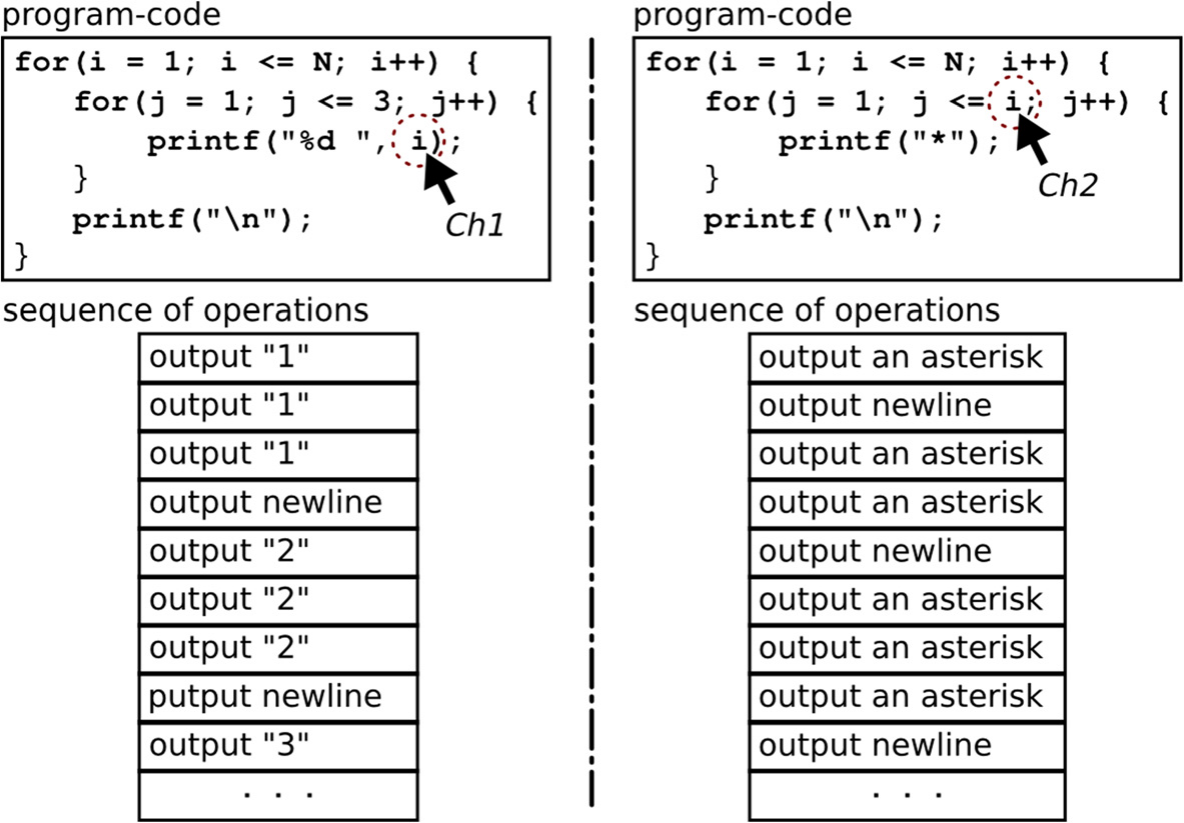
Program codes with Ch1/Ch2 and its operation sequence

A nested loop with Ch1 includes operations of varying representations at each iteration step of the outer loop—that is, for each inner loop. For example, the left side in Fig. 5 is the program code for outputting different values according to each iteration step of the outer loop. Conversely, a nested loop with Ch2 has a different number of operations corresponding to the inner loop according to each iteration step of the outer loop. For example, the right side in Fig. 5 is the program code where a different number of operations corresponds to the inner loop according to each iteration step of the outer loop.

We define “changing-step-contents type” as a type of nested loop with Ch1 and “changing-step-counts type” as a type with Ch2. The behavior of a nested loop can be classified into the following four classes based on the absence or presence of each characteristics:

- Without either Ch1 or Ch2—the operations corresponding to each inner loop and the number of them will never change.
- With Ch1—the operations corresponding to each inner loop will change in each outer loop step; however, the number will not.
- With Ch2—the number of operations corresponding to each inner loop will change in each outer loop step; however, the operations themselves will not.
- With both Ch1 and Ch2—both the operations corresponding to each inner loop and the number of them will change in each outer loop step.

In their packing series, learners must appropriately recognize the repetitiveness of an operation sequence as to whether it has these characteristics. Furthermore, to recognize the repetitiveness, learners must anticipate the regularity in the operation field based on the characteristics in the program code field. If learners can classify a nested loop code, they can reproduce the behavior of the nested loop based on the behavior pattern of the class. Moreover, by determining the behavior pattern of an algorithm in the coding, novice programmers can identify the characteristics to incorporate into the program code.

Based on these discussions, we attempt to teach students these characteristics, aiming to develop in them a better understanding of nested loops. Furthermore, we have implemented a function that involves requesting users to identify the type of nested loop provided in the program code field and whether it is the changing-step-contents type, the changing-step-counts type, both, or neither. For users who cannot provide an answer, LEPA2 suggests hints:

1. Hints related to where in the program code they should focus
2. Hints as to what characteristics they should be reading in the code revealed by the above hints
3. The characteristics that should have been read

### Our extended system

**Fig. 6:**
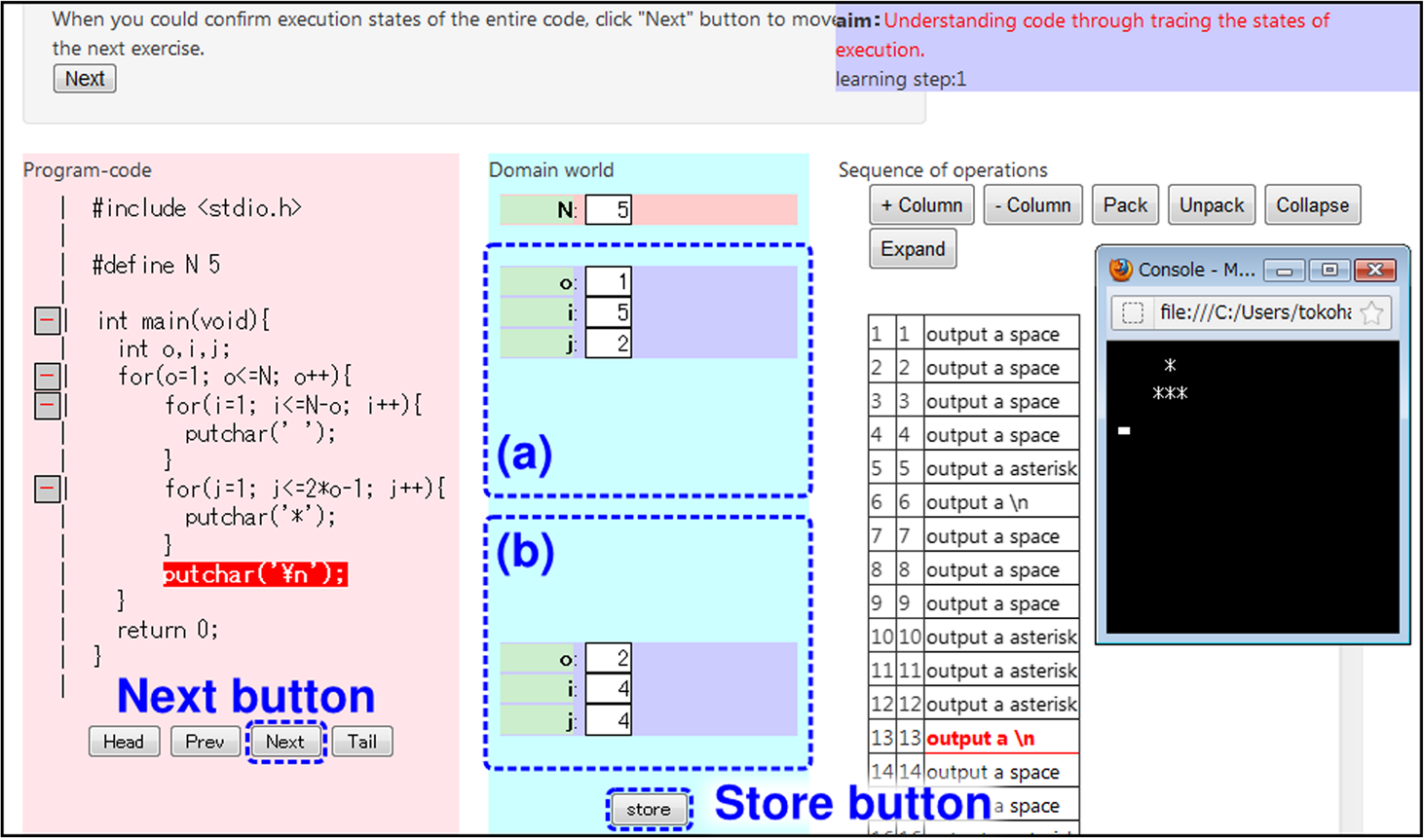
Ex1 on LEPA2

In this subsection, we present an overview of the proposed extended system, LEPA2. Although the external appearance of LEPA2 is similar to that of LEPA1 (Fig. 1), LEPA2 places more emphasis on learning nested loops than LEPA1. LEPA2 helps learners to understand based on the following scenarios:

Ex1—*Tracing*: First, learners trace the program code and understand the behavior and control flow of the entire program. Figure 6 provides an overview of the environment constructed by LEPA2 at this phase. If the learners push the “Next” button, LEPA2 performs the following actions:

- Highlights the statement that will be executed next
- Displays the status of the target domain world after executing the statement
- Highlights the operation corresponding to the statement (if one exists)

Moreover, if the learners click an operation in the operation field, LEPA2 highlights the statement in the program code field corresponding to the operation. If the learners push the “Store” button, LEPA2 copies the current status of the target domain world displaying in the area (b) into (a). This function support learners to observe the differences between the status of the target domain before and after executing specific statements or operations.

**Fig. 7:**
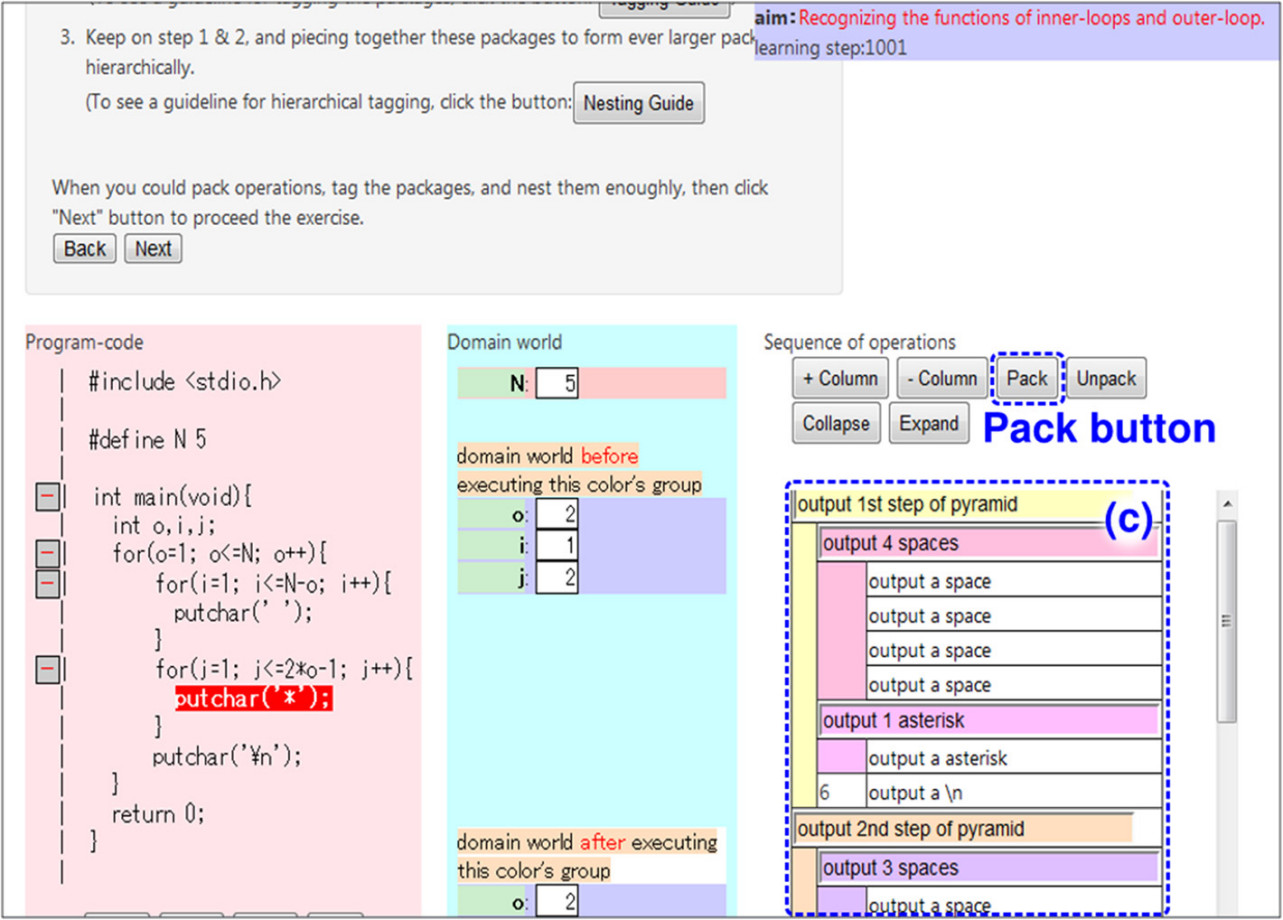
Ex2 on LEPA2

Ex2—*Abstraction*: Learners pack operations and recognize the abstract function of the package. Packing with nested structures helps learners to understand the nested structures of the entire program. Figure 7 provides an overview of environment constructed by LEPA2 at this phase. Learners find a certain sequence of operations that is making a change with a specific meaning in the target domain world, based on the findings in Ex1. The “Store” button is useful to fulfill this. Learners select the range of the sequence of operations in area (c) and pack the operations by clicking the “Pack” button. The resulting package is indented and put different colors on each other, and LEPA2 encourages learners to tag the package. Learners put the meaning of the changing into a natural-language phrase and input the phrase in the textbox set in the package.

**Fig. 8:**
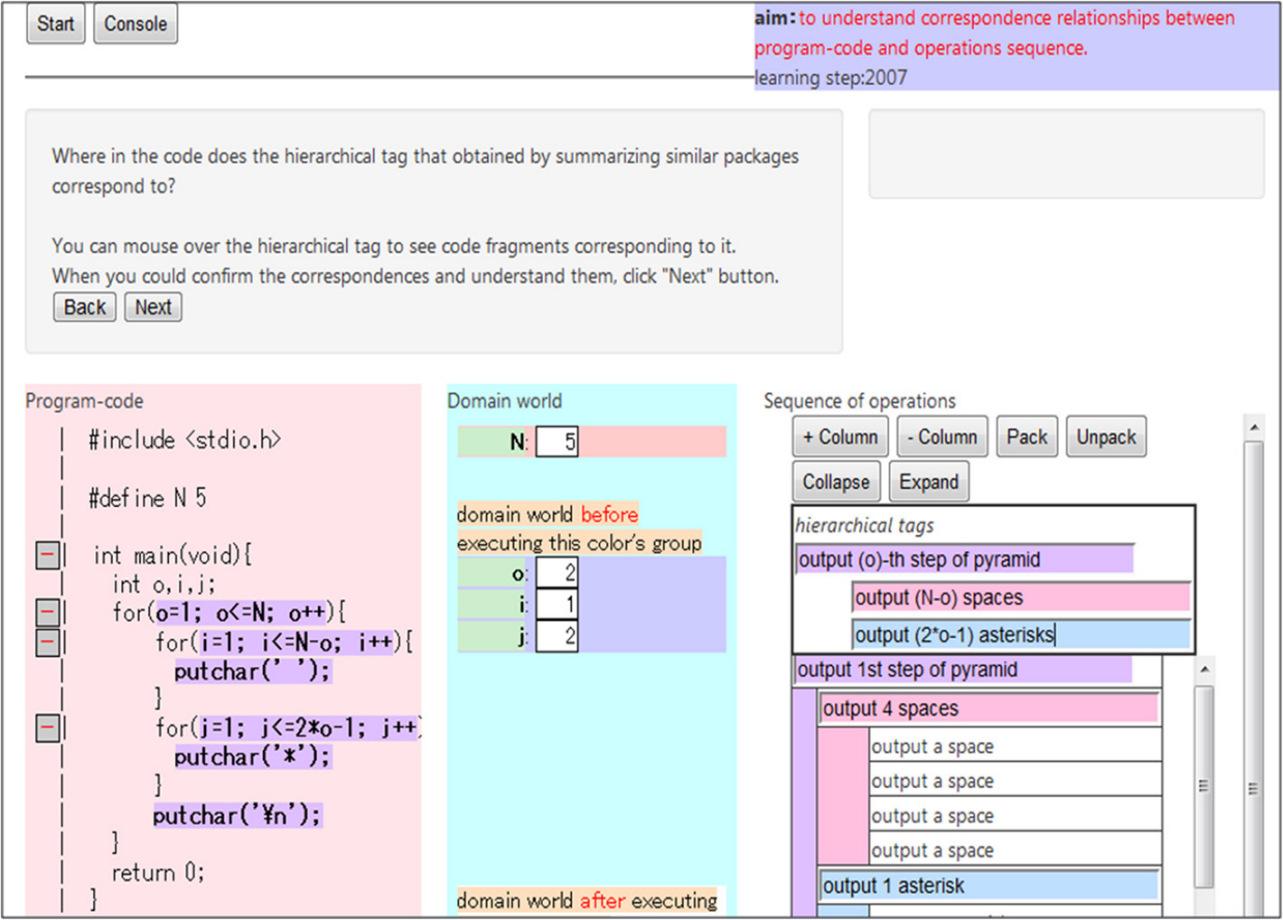
Ex3 on LEPA2

Ex3—*Generalization*: After completing the packing for all the operations, learners generalize the packages corresponding to the nested loops, following the guidance of LEPA2. LEPA2 generates templates for the general tag based on the approach described in the “Gap between the representations of generalization” section. Learners complete the template according to the resemblance between the structure of the generalized package and that of the program code. Figure 8 provides an overview of the environment constructed by LEPA2 at this phase.

In nested loops, the repetitiveness to be discovered during the generalization phase does not appear in the concrete operations. Rather, it appears in the tags to packages packed by the learner. We have implemented the following functions to notify the learner in Ex3:

- Auto-complete function to support creating repetitive packages by displaying the learner’s tagging history
- Function to support focusing on repetitive packages by displaying similar tags in the same color
- Function to support searching for the repetitiveness in the tags by hiding the concrete operations
- Function to support appropriately tagging the *n*th lap by providing a generalization template

**Fig. 9:**
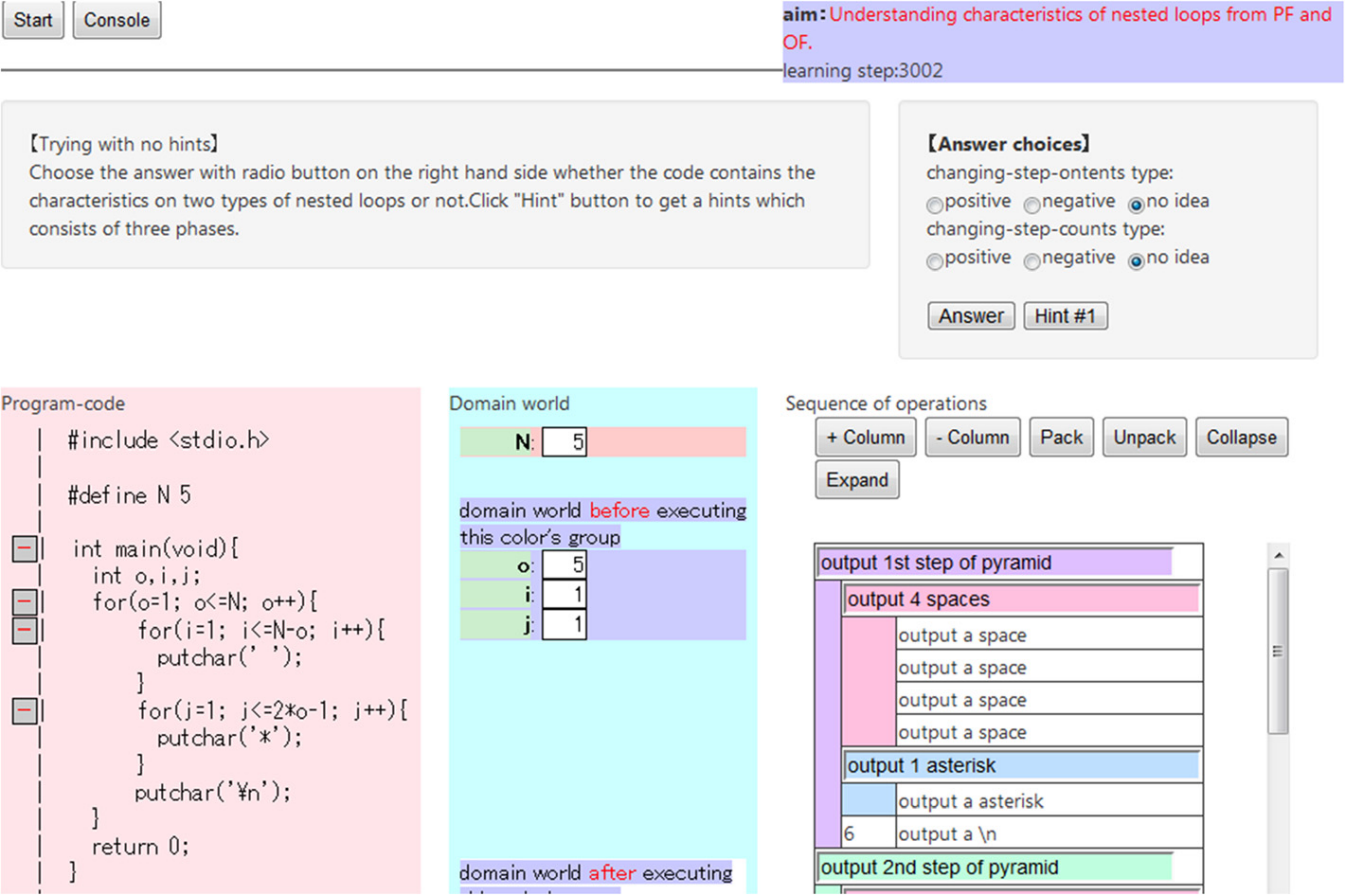
Ex4 on LEPA2

Ex4—*Observing characteristics of the nested loop*: Lastly, learners observe the characteristics of the nested loop in the program code field to increase their understanding of nested loops. LEPA2 requests learners to identify the characteristics of nested loops based on the approach described in the “Two characteristics in nested loop code” section. Figure 9 provides an overview of the environment constructed by LEPA2 at this phase.

## Educational practice

### Hypotheses on learning effect with the proposed system

We conducted exercise classes to understand nested loops with LEPA2 as described in the previous section. Our previous educational practice (Kogure et al. 2013) suggested that learning with LEPA1 had the following effects:

- Learners could acquire the skills of tracing values of variables in the entire program code through observing the statuses in the target domain field.
- Learners could understand nested structures in control flows and code descriptions through packing sequences of operations hierarchically.
- Tags and structures of packages of operation sequences could visualize each learner’s level of understanding and could provide information for better instructions to teachers.

The exercises practiced in our previous work consisted of tracing the program code with nested loops and packing sequences of operations corresponding to the code. The learning scenario described in the “Our extended system” section includes the same tasks as the previous one, further tasks that generalize the packages corresponding to the nested loops based on the guidance of LEPA2, and tasks that observe the characteristics of the nested loop. Consequently, learning with LEPA2 is expected to help learners to cultivate better understandings of nested loops. Through the educational practice in this work, we evaluate the following learning effects for LEPA2:

- Hypo1. The redeveloped function of package generalization will promote an understanding of the behavior and structure of a nested loop.
- Hypo2. The function that requests an answer to a question based on the characteristics of the nested loop will promote an understanding of the relationships between the implementation and behavior of a nested loop.

### Overview of the classroom practice

To verify the two hypotheses described above, we conducted an educational practice as a controlled experiment. We introduced LEPA2 in the experimental practice and did not introduce any learning support system in the controlled practice. There were 17 subjects in the entire experiment. In the experimental practice, two consecutive exercise classes were incorporated into a series of actual classes held in a university in Japan. The department holds two courses, Programming I and Programming II, for second-year students. Our experimental classes were conducted in the latter course. There were 12 participants in the experimental practice. All 12 participants were Business Administration majors, 20-year-old males, and had less than a year’s experience in programming. In the control practice, two consecutive exercise classes were held out of the actual classes. There were five participants in the control practice. All five participants had the same properties as the experimental group, that is, they were Business Administration majors, 20-year-old males, and had less than a year’s experience in programming. Table 1 presents a summary of the experimental and control classes.

**Table 1:** Summary of experimental and controlled classes

	Experimental	Controlled
Style of class	Lecture and exercise	Lecture and self-study
Learning time	90 min and after a week, 90 min	90 min and after 10 min, 90 min
Learning strategy	LEPA2	Traditional textbooks
The number of participants	12	5

For the experimental practice, the teacher who regularly taught the course lectured on single and nested loops for 1 h as a review in the first class. The lecture included an explanation of the characteristics of nested loops, as described in the “Two characteristics in nested loop code” section. At the end of the class, we conducted a 15-min pre-test to evaluate the students’ understanding of nested loops before using LEPA2. In the second class, we allowed the students to use LEPA2 to learn nested loops. The program for this exercise displayed a five-step pyramid with spaces, asterisks, and new lines. Before the exercise in the class, the teacher described the aims of the exercise and the environment provided by LEPA2. During the exercise, neither the teacher nor our team provided assistance to the students for understanding the program. We recorded screen videos of the student’s interactions with the environment. After the 60-min exercise, we conducted a 15-min post-test to evaluate the students’ understanding of nested loops after using LEPA2. In the beginning of the first class, we informed that the students would have pre- and post-tests but the resultant scores of both test would not affect the course grades.

The programs used in the pre- and post-tests were different. However, the questions were almost identical. Question 1 (Q1) asked for the execution results expected from tracing the entire program by hand, including the nested loop. Question 2 (Q2) asked for the code fragment in the control of the inner loop with the execution results provided; question 3 (Q3) asked for the same for the outer loop. Question 4 (Q4) asked for the characteristics of the nested loops expected to appear in the program code, given the execution results only. Q1, Q2, and Q3 were designed to verify Hypo1; Q4 was designed to verify Hypo2. Table 2 presents a summary of each question in the pre- and post-tests.

**Table 2:** Summary of questions in pre- and post-tests

	Content of question	Maximum point
Q1	Describe the execution result of the program code.	3 (3 pts × 1)
Q2	Modify the control statement of the inner loop so as to output the indicated execution result.	9 (3 pts × 3)
Q3	Modify the control statement of the outer loop so as to output the indicated execution result.	3 (3 pts × 1)
Q4	What characteristics should be written in program code to output the indicated execution result.	48 (3 pts × 16)

For the control practice, students were first given the same 1-h lecture on nested loops as the experimental group, including an explanation of the characteristics of nested loops. We subsequently conducted the 15-min pre-test. Then, they studied nested loops using textbooks and the lecture material, without using LEPA2. Before studying, the teacher explained that the aim of the study was to understand nested loops using a sample program. The sample program was identical to the one in the experimental practice. The teacher also explained that, in particular, they should study tracing the program. After a 1-h study period, we conducted the 15-min post-test. Both pre- and post-tests were the same as those given to the experimental group.

Table 3 presents the differences in the average score for each question between the pre- and post-tests for both groups. We graded each question in the tests as follows: Q1 was worth three points; Q2 was worth nine; Q3 was worth three points; and Q4 was worth 48. There was a tendency for the experimental group’s scores to improve significantly as indicated by the differences between pre- and post-tests, whereas in the control group they did not.

**Table 3:** The differences in the average scores between the pre- and post-tests

	Q1	Q2	Q3	Q4
Experimental	0.83	0.75	0.50	1.50
Control	1.20	0.20	−0.60	−5.70

We think that the improved results for the control group with regard to Q1 were a consequence of emphasizing tracing as the aim of the study. It is likely that the control group applied a significant portion of their time tracing. Therefore, we consider this group to have an advantage over the experimental group, whose study consisted of four stepwise exercises.

The experimental group had an interval of 1 week between pre- and post-tests because of schedule restriction. This was because we incorporated the classroom practice into a series of actual classes. This interval is not short and consequently allowed the experimental group to learn out of class with textbooks and course notes. Although the influences of this possibility on the score of the post-test must be considered, it is difficult to quantify them. However, we infer that the influences are practically trivial because all the experimental subjects had learned nested loops in Programming I classes held in an earlier semester. If the subjects had understood nested loops in that earlier class or by out-of-class learning, they would have tended to score high in the pre-test. Note that we conducted classes for nested loop again because the lack of student’s understanding of nested loop hinders the exercise in Programming II. There was no actual tendency to score high; thus, we consider the influence of out-of-class learning as a negligible factor.

If we conducted both the pre- and post-tests in the same class session, the subjects would have a week interval between the pre-test and the classroom lecture because of the schedule restriction. In this case, the possibility of forgetting lecture contents would influence the score of the pre-test.

### Evaluation based on learner’s activities

We carefully reviewed the footage recorded from each student in the experimental group to analyze the learning effects in additional detail. Thereby, we found that the interactions with the learning environment provided by the proposed system differ significantly by student. For some activities made by using functions we expected to use, we classified the students into those who performed the activity (the positive group) and those who did not (the negative group). We rejected the activities that have greater than double times difference between the positive and negative groups to reduce the bias between the numbers of the both groups. Consequently, we focused on the following four activities:

- A1. The student consumed sufficient time (more than 3 min) tracing the program (Ex1).
- A2. The student packed all operation sequences corresponding to the inner loops and tagged all of the packages (Ex2).
- A3. The student formulated a general tag provided in the form of a template, using the guidance function from the outer loop generalization (Ex3).
- A4. The student used the function to observe the characteristics of nested loops (Ex4).

In A1, 3 min is the time we supposed to take to trace the entire program code with observing three fields. In actual exercise, there was bipolarization between the time to trace by the positive group, who take much more than 3 min, and the time to trace by the negative group, who take less than 1 min.

We consider these activities to lead to the following learning effects:

- A1 advances an understanding of the behavior of an entire nested loop from which the student will acquire tracing skills.
- A2 promotes an understanding of the step contents in the inner loops from which the student will understand the structure of a nested loop.
- A3 assists the understanding of the step contents in the outer loop from which the student will acquire the skills required to generalize concrete operations.
- A4 leads to an understanding of the characteristics of nested loops from which the student will understand the relationships between the behavior and the characteristics in the program code.

We expect that students performing these activities will improve their marks as follows: students who performed A1 will score better on Q1; those who performed A2 will score better on Q2; and likewise for those performing A3 and A4 for Q3 and Q4, respectively.

Table 4 presents the differences in the average scores for each question between the pre- and post-tests in the positive and negative groups for A1 through A4. By performing an independent *t* test, if the difference between the negative and positive groups is statistically significant such that the progress level reached *p* = 0.05, we placed an asterisk next to the number. As expected, each positive group demonstrated significant progress for their marks on the corresponding question.

**Table 4:** Differences in the average scores for positive and negative groups

		Number of students	Q1	Q2	Q3	Q4
A1	Positive	7	1.43*	1.57	1.29*	
	Negative	5	0.00	−0.50	−0.75	
A2	Positive	5	0.80	2.60*	0.60	
	Negative	7	1.00	−0.67	0.50	
A3	Positive	4	1.17	1.67	1.50*	
	Negative	8	0.60	−0.20	−0.60	
A4	Positive	4				2.63*
	Negative	8				−0.75

A1 to A4 are the four learning activities; *p < 0.05

We must consider that a statistically insufficient number of the subjects may influence the accuracy of verification. Our evaluation results do not have sufficient reliability because we could not procure a sufficient number of subjects in the classroom practice. The course we selected for our class is optional for the faculty, and the number of applicants varies from year to year. Whereas practice in actual classes provides practical learning information, the number of subjects is related to the number of applicants. However, we believe that continuous practice will suppress this matter. The progress with regard to Q1, Q2, and Q3 suggests in favor of Hypo1 and that of Q4 for Hypo2. Hence, although preliminarily, the results suggests that the proposed system results in an improved understanding of nested loops provided the user performs the expected activities. We must consider that a statistically insufficient number of the subjects may influence the accuracy of verification. However, we believe that continuous practice will suppress this matter.

## Related works

In general, programming students learn algorithm, code readings, and coding in turn. They attend a lecture and receive algorithm instruction from their teacher. Then, they reproduce the behavior of the algorithm using certain input data and produce a sequence of concrete operations that represents the behavior of the algorithm. Subsequently, they abstract sequences of operations, grasp the relationship between the abstracted operations and the program code, and consequently understand the entire program code. Finally, they perform a coding exercise to confirm their understandings.

Thus far, several intelligent tutoring systems have been developed to support programming learners. These include RoboProf (Daly & Horgan, 2004), JITS (Sykes & Franek, 2003), J-LATTE (Holland et al. 2003), and BITS (Butz et al. 2006). Moreover, several learning support systems based on visualizing algorithms have received attention, including TRAKLA2 (Malmi et al. 2004), Jeliot 3 (Moreno et al. 2004; Čisar et al. 2011), and ViLLE (Rajala et al. 2008). These systems can be classified from the standpoint of the tasks required for understanding an algorithm or program code and tend to support one or the other. We believe that an attractive learning target can be found in the gap between these two tasks. These systems, however, do not offer a suitable means for bridging the gap.

Learners who have a proper understanding of an algorithm can reproduce its behavior with concrete data. A sequence of operations in LEPA is a sequence of natural-language descriptions representing the algorithm’s behavior. Hence, the operation sequence can be regarded as an externalization of the learner’s understanding of the algorithm. Other existing systems visualize the relationship between the program code and its target domain world. LEPA provides this as well and furthermore visualizes the relationship between a sequence of operations and its target domain. It also visualizes the correspondence of a code fragment to its operation. We expect that the visualization of these three fields and the relationships among them contributes to bridging the gap between the two tasks.

When a program-comprehension task is assigned to a programmer, the procedure for reading the code is normally a dual process: first, recognizing the function of groups of statements and then piecing together these chunks to form ever-larger chunks (Shneiderman & Mayer, 1979). Programmers proceed through these steps hierarchically until the entire program is understood. LEPA offers a function to support learners in their endeavor to envisage behavior similar to that in the operation field. Learners can learn an entire series of code-reading process using LEPA by this function.

Recently, learning support systems with an integrated development environment have been developed, aiming to support the entire programming exercise (Đanić et al. 2011; Gerdes et al. 2012; Neve et al. 2012). These systems do not focus on the code-reading stage. Consequently the learners only externalize their understanding of the algorithm and program code by coding. Learners and teachers cannot determine the cause of a learner’s lack of understanding: lack of algorithm understanding, program code understanding, or coding skills. Further, we can identify systems based on externalization with other methods than coding (Cooper et al. 2012). However, they are intended to support younger learners and cannot directly support users to read and write the program code. More discussions are required to introduce these systems into current programming education at the university level.

In contrast, LEPA encourages the learner to abstract groups of statements in the program code and to externalize the abstracts in the form of tags. Learners trace the program code, observe the changes in the target domain field, pack the operations in the operation field according to the observation, and tag the packages according to the function. The learner’s package structure is the product of externalization with a series of these activities. LEPA does not include correct package structure solutions, hence never provides a solution, and never leads the learner to the correct solution. Thus, teachers can consider the package structures as the learner’s understandings externalized by the proposed system. As will be shown in the next section, we consider that these functions improve the quality of programming education.

There are systems available that target nested loops, including AlgoTutor (Yoo et al. 2012) and the tutoring system developed by Dancik and Kumar (2003). However, it is not clear how they lead learners to understand or acquire the fundamental concepts described in the “Introduction” section. If any chunk corresponds to an iteration such as a loop, it is often the case that the learner’s recognition involves generalizing with variables. LEPA2 bridges the gap between hierarchical structures of chunks in a sequence of operations and those of descriptions in the program code and aims to improve the learning supports for nested loops by elaborating the strategy of a hierarchical procedure for reading code.

## Discussion

In this section, we discuss the contribution of the proposed system to learning programming. The correlations suggested by the classroom practices described in the “Educational practice” section and in our previous work (Kogure et al. 2013) can be summarized as follows:

- Learning with the proposed system can contribute to acquiring fundamental skills to understand programs.
- Tags and structures of packages of operation sequence can visualize each learner’s level of understanding and can provide information for better instruction to teachers.

In traditional education for programming in the code-reading stage, the teacher explains the functions and the behavior of the sample code in the textbooks. Learners require a detailed understanding of the sample code because it is typical of each learning target. However, code reading is a difficult learning activity to externalize achievements. Teachers attempt to understand indirectly the learner’s level of understanding by exercises in writing similar code or tracing values of the variables. It is not obvious that the achievements of these exercises reflect appropriately the level of understanding of the sample code.

**Fig. 10:**
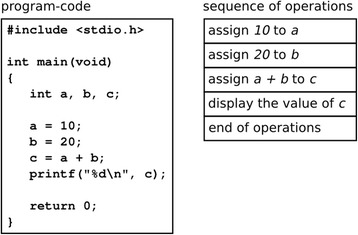
Program code from textbooks and its operation sequence

**Fig. 11:**
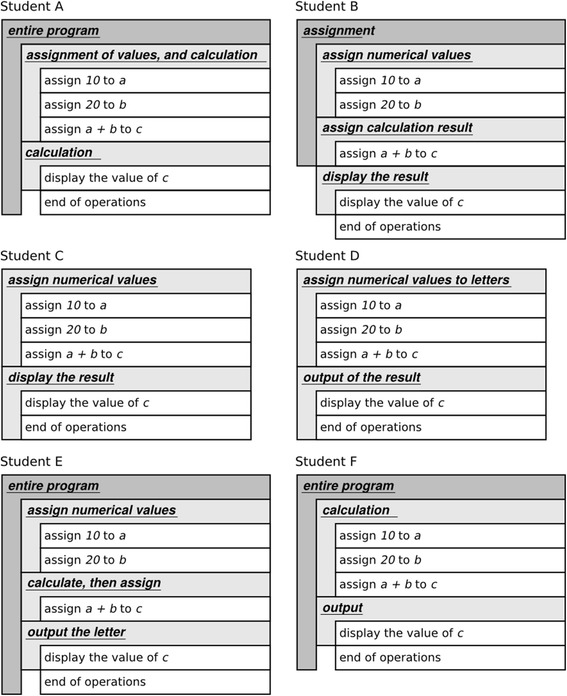
Package structures generated by six students

As described in the previous section, learners with the proposed system externalize their understandings in observable forms for themselves and their teachers through packing, generalizing, and tagging operations in the operation field. To confirm the validity of the proposed system’s externalization, we invited six students to read code with the proposed system as a pilot experiment. The students had not attended the practices and experiments described in the “Educational practice” section. The code was from an early section of our textbooks. Figure 10 presents the code and the corresponding sequence of operations. Figure 11 presents the packages generated by the students. There are several observations of the students’ understandings:

- Some students packed the operations assigning constants and operations assigning calculated values separately, whereas others combined them into a single package.
- Some students recognized that data is stored to a letter rather than a variable.
- Some students recognized the settings of the values into variables *a*, *b*, and *c* as calculations rather than assignments.

The intent of the sample code was to calculate and display the value 10 + 20. A lack of understanding of intention could possibly yield the first and third developments. Students with recognition as in the second development could possibly not appropriately grasp the concept of variables.

We consider that the proposed system improves the quality of programming education based on these observations. In the classroom exercise with the proposed system, teachers can observe the status of the students’ code reading by walking around the class. Because the students are externalizing their understanding using the proposed system, the teachers can grasp the students’ understandings by observing operation field in each student’s environment. If there is a misunderstanding regarding package structures and tags generated by a certain student, the teacher can offer the student high-quality instructions by separate interaction. If the teacher identifies a tendency of the class to misunderstand as a whole, they can provide the supplemental instruction in the form of classroom lectures. Enabling teachers to choose the appropriate style of instruction improves the quality of education and leads students to a superior understanding of the program code.

For example, in the results of the pilot experiment, there is a student (student D) suspected of not appropriately understanding the concept of variables. There is also a tendency of the class not to distinguish between using a constant number from reference variables. This information provided by LEPA allows teachers to make a decision as to the required instruction, especially to make opportunity discovery. In the pilot experiment, we did not evaluate the contribution degree. We plan to conduct further experiments to obtain more information regarding this.

In programming education, there are minimal reports available on the learning effect of code reading. In our opinion, this is caused by the difficulty in externalizing learning outcomes. As indicated in Fig. 11, package structures in the proposed environment provide useful information to teachers regarding a learner’s understanding. Thus, we consider that the proposed system not only directly supports learners to cultivate a superior understanding of the program code but also indirectly by improving teacher’s performance.

## Conclusion

In this paper, we described our learning support system and classroom practices with the proposed system for an improved understanding of nested loops. Nested loops are an appropriate target for learning the fundamental skills of programming. We believe that learning support systems efficiently and effectively contribute to an understanding of fundamental programming concepts and the acquisition of the skills required to utilize them.

In the classroom practice with LEPA1, we determined that some students had experienced a learning impasse. We attempted to address this with two supporting approaches: bridging the gap between generalization structures in the program code and their corresponding operations and enabling learners to predict the behavior of nested loops by observing two characteristics in the code. For the former approach, we developed a function for generalizing packages by maintaining an explicit hierarchy. For the latter, a function allowing learners to observe the characteristics of nested loops was provided.

We conducted a controlled experiment to evaluate in detail the effect of LEPA2 with the abovementioned functions incorporated. In the experimental practice with 12 students forming an experimental group, we incorporated LEPA2 in an actual classroom. In the controlled experiment with five students forming a control group, we evaluated learning effects using traditional teaching materials such as textbooks. The evaluation results based on the scores calculated from a pre- and post-tests suggest that there was a correlation between using LEPA2 and an understanding of nested loops although preliminarily.

Package structures and tags generated in learning with the proposed system can be regarded as an externalized learner understanding that is observable by teachers. Feedback provided by these is useful information for teachers’ instruction. Consequently, qualitative improvements of instruction are expected. Hence, we consider that the proposed system directly and indirectly supports learners to read the program code and cultivate a better understanding of the program code.

We can highlight two limitations of the proposed works derived from classroom practices incorporated into a series of actual classes. First, our evaluation results do not have sufficient reliability because we could not procure a sufficient number of subjects in the classroom practice, as described in the “Evaluation based on learner’s activities” section. We expect that continuous practice will overcome this matter. We plan to continue to conduct classroom practices using the proposed system in the future.

Furthermore, we must allow a 1-week interval between pre- and post-tests in experimental practice because of the schedule restriction. We infer that the influences of this are minimal, as described in the “Overview of the classroom practice” section. It is difficult or almost impossible to quantify and eliminate these factors. We continue to deliberate from various viewpoints such as conducting educational practice outside of actual classes and constructing an efficient learning scenario where we can evaluate learning effects more accurately during a class.

In future work, we will consider how the proposed system contributes to improve education for programming, as described in the “Discussion” section. We will collect package structures from the operation fields packed by learners through further classroom practice and evaluate the relationships between the students’ understandings and those structures. Moreover, we will develop an evaluation method to measure how the proposed system contributes to the decision-making on teachers’ instruction. Furthermore, we will continue to develop a desirable learning scenario that naturally causes the learners to perform the appropriate learning activities expected by the proposed system.
